# Periplasmic Nanobody-APEX2 Fusions Enable Facile Visualization of Ebola, Marburg, and Mĕnglà virus Nucleoproteins, Alluding to Similar Antigenic Landscapes among *Marburgvirus* and *Dianlovirus*

**DOI:** 10.3390/v11040364

**Published:** 2019-04-20

**Authors:** Laura J. Sherwood, Andrew Hayhurst

**Affiliations:** Disease Intervention and Prevention, Texas Biomedical Research Institute, 8715 W. Military Dr., San Antonio, TX 78227-5302, USA; lsherwood@txbiomed.org

**Keywords:** peroxidase, APEX2, VHH, sdAb, nanobody, Filovirus, Ebola, Marburg, nucleoprotein, nanoluciferase

## Abstract

We explore evolved soybean ascorbate peroxidase (APEX2) as a reporter when fused to the C-termini of llama nanobodies (single-domain antibodies, sdAb; variable domains of heavy chain-only antibodies, VHH) targeted to the *E. coli* periplasm. Periplasmic expression preserves authentic antibody N-termini, intra-domain disulphide bond(s), and capitalizes on efficient haem loading through the porous *E. coli* outer membrane. Using monomeric and dimeric anti-nucleoprotein (NP) sdAb cross-reactive within the *Marburgvirus* genus and cross-reactive within the *Ebolavirus* genus, we show that periplasmic sdAb–APEX2 fusion proteins are easily purified at multi-mg amounts. The fusions were used in Western blotting, ELISA, and microscopy to visualize NPs using colorimetric and fluorescent imaging. Dimeric sdAb–APEX2 fusions were superior at binding NPs from viruses that were evolutionarily distant to that originally used to select the sdAb. Partial conservation of the anti-*Marburgvirus* sdAb epitope enabled the recognition of a novel NP encoded by the recently discovered Mĕnglà virus genome. Antibody–antigen interactions were rationalized using monovalent nanoluciferase titrations and contact mapping analysis of existing crystal structures, while molecular modelling was used to reveal the potential landscape of the Mĕnglà NP C-terminal domain. The sdAb–APEX2 fusions also enabled live *Marburgvirus* and *Ebolavirus* detection 24 h post-infection of Vero E6 cells within a BSL-4 laboratory setting. The simple and inexpensive mining of large amounts of periplasmic sdAb–APEX2 fusion proteins should help advance studies of past, contemporary, and perhaps Filovirus species yet to be discovered.

## 1. Introduction

Although horseradish peroxidase (HRP) is a legendary reporter enzyme, it is very fickle to produce high levels of soluble protein in *E. coli*, and is still commercially isolated from the roots of the horseradish plant for purification and chemical conjugation to polyclonal and monoclonal antibodies. Peroxidase antibody conjugates are typically used as secondary antibodies to allow the visualization of antigens within cells and tissues, on Western blots and ELISAs, with a variety of chemiluminescent, colorimetric, and fluorescent substrates available. Advances in recombinant antibody technology have enabled many laboratories to produce the antigen binding portions of a given antibody in *E. coli*, immortalizing the clone and enabling the inexpensive production of genetic fusions with reporter enzymes of choice. Bypassing chemical conjugation ensures an antibody:reporter stoichiometry of 1:1 and also eliminates conjugation chemistry destroying or occluding the antigen binding site, which can comprise a substantial portion of smaller recombinant antibody formats [1]. Unfortunately, the complexity of HRP has hindered the efficient production of functional antibody–enzyme fusions in *E. coli* to date.

HRP has four disulfide bonds, two calcium ions and a central haem which are essential for the folding process [2,3]. While incremental advances have been made in HRP yields through providing disulfide isomerases in trans [4] and cis [5], low yields and/or low catalytic activities have driven efforts towards other hosts including yeasts [6] and costly mammalian cell culture [7]. The need for disulfide bonding and haem mandates secretion into an oxidizing environment for the production of correctly folded active protein. The outer membrane of *E. coli* is typically porous to molecules of around 650 Da and smaller by virtue of large porins [8] and since hemin, a convenient source of haem, is 652 Da, there is no diffusion limitation. In contrast, employing a non-secreted expression route to the reducing cytosol of *E. coli* enveloped by a more impermeable inner membrane that limits haem generates large quantities of insoluble protein which subsequently requires lengthy refolding regimes.

During our own struggles to overexpress soluble HRP in the periplasm, we wondered whether the recently described soybean ascorbate peroxidase derivative APEX2 [9] would be easier to produce, since the enzyme lacks disulfide bonds and calcium, though it does still possess haem [10]. APEX2 was derived through successive rounds of directed evolution for in situ proximity labelling and electron microscopy within mammalian cells, following the intracellular expression of APEX2 fusions with a target protein of interest [9,11,12]. APEX2’s substrates include 3, 3’-diaminobenzidine (DAB), a widely used and inexpensive precipitating chromogen for Western blotting and immunocytochemistry, and Amplex^®^UltraRed, a soluble chromogen also with fluorescence capability. We hypothesized that if we could express sufficient APEX2 in the periplasm, the platform would deliver sdAb–APEX2 fusion proteins to enable one-step staining for the presence of virus antigens on blots and in cells using light and fluorescence imaging.

Our model recombinant antibodies were semi-synthetic llama single domain antibodies (sdAb) previously selected on live *Marburgvirus marburgvirus* (MARV Musoke) and *Zaire Ebolavirus* (EBOV Kikwit) preparations [13,14]. All of our sdAb were shown to be reactive against the C-terminal region of nucleoprotein (NP), the major internal component of the ribonucleocapsid enveloping the viral RNA genome. SdAb are 1/10th the size of an IgG and favor concave epitopes that may not necessarily be available to conventional antibodies with more planar paratopes [15]. Thus, sdAb can be particularly useful for targeting pathogens [16,17]. For the long-term recognition of RNA virus epitopes, this is important, since such “cryptic” epitopes tend to be highly conserved and reduce the likelihood of a particular antibody becoming obsolete through viral evolution. Indeed, structural analysis has since revealed that our anti-Marburg sdAb favored a hydrophobic basin at the NP C-terminus that is completely conserved among MARV and Ravn virus (RAVV) isolates to date [18]. Since NP is an internal antigen, it is even less prone to drift as a result of B-cell surveillance, though it would still expect to be under T-cell surveillance.

Typically, sdAb, like peroxidases, are also targeted to the *E. coli* periplasm since they have one or more disulfide bonds. Most sdAb historically have also been selected using g3p based phage display and may well require a free N-terminus to preserve full antigen binding capacity, and signal peptidase cleavage during periplasmic export guarantees this. Periplasmic expression has also enabled us to assemble tandem dimers to capitalize on the benefits of avidity and increase sensitivity of both MARV and EBOV NP recognition in vitro and enable a novel antiviral crosslinking strategy [19].

Herein, we generated gene fusions of our monomeric and dimeric sdAb with APEX2 downstream, assessed their ability to be produced in the periplasm of *E. coli*, and then employed them in detecting filovirus NP using a variety of strategies. Since we had previously claimed our sdAb would be useful for detecting *Marburgvirus* species yet to emerge based on our structural studies [18], we capitalized on the recent discovery of a new Filovirus genome in China [20] (Mĕnglà virus, MLAV, belonging to a new genus *Dianlovirus*) to show our sdAb could indeed recognize the putative NP product. Interrogation of the differential recognition of MARV and MLAV by three sdAb-based on existing crystal structures and predictive modelling suggest similar overall architectures for the NP C-terminal domains. Since each 0.5 L expression culture of sdAb–APEX2 construct yields enough material for tens of thousands of assays for pennies, and the plasmids are immortalized in silico and cryopreserved these reagents may find utility both here and abroad.

## 2. Materials and Methods

### 2.1. sdAb–APEX2 Expression Vectors

The gene encoding APEX2 was obtained as a gBlocks^®^ sequence (Integrated DNA Technologies, Coralville, IA, USA). The sequence was based upon pCDNA3 APEX2-NES [9] available on the Addgene website (Watertown, MA, USA) deposited by the Ting laboratory. The design added a FLAG tag (Asp, Tyr, Lys, Asp, Asp, Asp, Asp, Lys) to the N-terminus minus the initiating Met, exchanged the single Cys codon at position 32 for a Ser codon, and silently deleted internal *Hin*dIII and *Pst*I sites. The N-terminal flank also encoded an *Nco*I site to allow in-frame fusion with the *pel* B leader sequence of pecan73 [21] for periplasmic secretion, while the C-terminal flank encoded a *Not*I site to allow in-frame fusion with the resident His^6^ sequence which is followed immediately by a termination codon. Clones were isolated in XL-1 Blue following standard cloning methods, mapped and the junctions plus incoming gene were sequenced.

Fusion protein vectors were subsequently made by first amplifying the APEX2 Cysdel gene using primers encoding an N-terminal *Not*I sequence (Ala, Ala, Ala, Gly, Lys, Ser…) and a C-terminal Gly^3^His6 tag plus *Hin*dIII sequence encoding a TAA termination codon, and inserting the fragment downstream of an anti-MARV NP sdAb (sdAb B). The resident sdAb was subsequently replaced with monomeric anti-EBOV NP sdAb ZE, monomeric anti-MARV sdAb A or dimeric versions of sdAb ZE and sdAb A from biotin acceptor phage display vector pecan126 [21] via *Nco*I and *Not*I. Herein, sdAb ZE will be referred to as sdAb E for simplicity.

### 2.2. Production and Purification of APEX2, sdAb–APEX2, and sdAb Proteins

Plasmids were mobilized into *E. coli* Tuner + pRARE for production and were grown in 50 mL starter cultures of terrific broth (TB) plus 2% glucose at 30 °C overnight with ampicillin (200 µg mL^−1^) and chloramphenicol (30 µg mL^−1^) in 250-mL Bellco baffled flasks. For APEX2 and APEX2 fusions, a stock solution of hemin (Sigma, St. Luois, MO, USA) was prepared by dissolving 32.5 mg in 500 µL 1.4 M ammonium acetate water (Sigma) and vortexing, which was then added to 450 mL glucose free TB while swirling in 2500-mL Bellco baffled flasks. The saturated overnight cultures were poured in to the large flasks (to yield a 1 mM final concentration of hemin) and were shaken for 3 h at 25 °C. Expression was induced by the addition of IPTG to 1 mM for 3 h at 25 °C, the cells pelleted, drained, and weighed. Cells were osmotically shocked [22] by resuspension in 14 mL ice-cold 0.75 M sucrose in 100 mM Tris-HCl pH 7.5, with the addition of 1.4 mL 1 mg mL^−1^ hen egg lysozyme (Sigma), followed by the drop-wise addition of 28 mL 1 mM EDTA pH 7.5 and swirling on ice for 15 min. A volume of 2 mL 0.5 M MgCl_2_ was added, swirling continued for 15 min and the cells were pelleted. The 45 mL supernatant (osmotic shockate) was mixed with 5 mL 10× IMAC (immobilized metal affinity chromatography buffer—0.2 M Na_2_HPO_4_, 5 M NaCl, 0.2 M imidazole, 1% Tween-20, pH 7.5), followed by 0.5 mL High Performance Ni Sepharose (GE Healthcare, Pittsburgh, PA, USA) and the suspension was gently mixed on ice for 1 h. The resin was pelleted at 3000 rpm for 5 min (Beckman Allegra 6R swing out rotor, Brea, CA, USA) and washed twice with 50 mL 1× IMAC solution before elution with 2 mL 0.5 M imidazole in 1× IMAC buffer, pH 7.4 in Poly-Prep^®^ columns (Bio-Rad, Hercules, CA, USA). The proteins were concentrated in Amicon 10 kDa ultrafiltration devices (Millipore, Billerica, MA, USA) to 200 µL for separation by size exclusion chromatography (SEC) on a Superdex Increase 10/300 GL column (GE Healthcare) operating in phosphate buffered saline (PBS). Preparations were made to 50% glycerol and aliquoted for long-term storage at −80 °C. The proteins were quantified by UV adsorption and analyzed by SDS-PAGE and Coomassie blue staining for impurities.

### 2.3. Recombinant NP Genes

Human codon-optimized genes encoding NP from MARV Musoke 1980, EBOV Kikwit 1995, SUDV Boniface 1976, RESTV Reston 1989, TAFV 1994 and BDBV 2007 have been described previously [14]. All genes were cloned into puma2 an hCMV intron A SV40 ori expression vector described previously [18] except SUDV NP which required intronless expression in puma4, a derivative of the hCMV adenovirus tripartite driven construct puma1 [19] with a synthetic mutant woodchuck hepatitis virus posttranscriptional regulatory element [23] inserted downstream between the *Not*I and *Afl*II sites. The MLAV NP sequence was derived from Genbank KX371887.2, ordered as a human codon-optimized gBlocks^®^ sequence and also mobilized to puma2 via unidirectional *Sfi*I cloning.

### 2.4. Cytochemical Staining of Cells Transiently Expressing Recombinant NP

Human embryonic kidney (HEK) 293T cells (ATCC, Manassas, VA, USA) were grown in Dulbecco’s modified Eagle’s medium (DMEM) with 4.5 g L^−1^ glucose, l-glutamine, sodium pyruvate (Corning cellgro), 5% fetal bovine serum (Corning, Corning, NY, USA) and penicillin/ streptomycin (complete medium) at 37 °C and 10% CO_2_ with humidity. Cells were seeded at 7.5 × 10^4^ in 300 µL per chamber on 8-well µ-slides (ibidi, Fitchburg, WI, USA) 18 h prior to transfection. Then, 125 ng miniprep DNA (approx. 1.25 µL) and 500 ng linear polyethylenimine (PEI, 1 mg mL^−1^, approx. 0.5 µL) were mixed in 15 μL serum-free DMEM to make a final volume of 30 µL, allowed to complex for 20 min at room temperature and then added to cells. After 24 h, slides were washed with warm serum-free DMEM twice. Slides were fixed with 10% formalin for 1 h at 4 °C. Slides were washed three times with PBS before permeabilizing with 0.1% Triton X-100 in PBS for 10 min. Slides were washed 3 times with PBS and blocked with 2% BSA, 0.05% Tween-20 in PBS for 1 h. The block was removed and then cells were probed with 10 nM APEX fusion proteins in 200 µL 2% BSA, 0.05% Tween-20 in PBS for 15 min. Cells were then washed 3 times with PBS 0.1% Tween-20 (PBST), then twice with PBS and developed with metal enhanced DAB tablet solution (Sigma) for 1 min before washing with PBS to stop the reaction.

For fluorescent staining, cells were probed with 10 nM of sdAb–APEX fusions and development was with 200 µL PBS containing 50 µM Amplex^®^UltraRed and 10 mM H_2_O_2_ for 30 min. Counterstaining was with Hoechst for 10 min followed by 2 PBS washes. Slides were viewed using an Eclipse Ti confocal microscope (Nikon, Tokyo, Japan) with NIS Elements Imaging Software employing a 590 nm emission filter for Amplex™ UltraRed visualization. ImageJ within Fiji was used to process images. Microscope and image processing settings were kept constant within anti-EBOV or anti-MARV to enable side-by-side comparison of monomers and dimers.

### 2.5. Small-Scale Recombinant NP Expression and Western Blotting

Lysates for Western blotting were generated by small-scale PEI transfections of the repertoire of NP plasmids or a β-galactosidase control plasmid. HEK 293T cells were seeded at 7.5 × 10^5^ cells per well in a 6-well plate in 3 mL complete medium at 16–18 h prior to transfection. A volume of 12.5 μL DNA (approx. 1.5 µg) and 5 μL linear PEI (1 mg mL^−1^) were combined and equilibrated for 10–15 min at room temperature in 300 μL serum-free DMEM prior to being carefully added to the cells. At 48 h post-transfection, cells were washed gently with 1 mL warm serum-free DMEM and then collected in 300 μL 1× Laemmli sample buffer with reducing agent and briefly sonicated. All samples were stored at −20 °C before further processing. Samples were heated at 100 °C for 5 min before 2.5 µL was loaded on to SDS-PAGE Laemmli gels. Gels were semi-dry transferred to Immobilon P and the membrane was blocked in Carnation 2% non-fat dried milk in PBS (MPBS) for 1 h prior to probing with 10 nM of the various sdAb–APEX2 fusions for 1 h. Following washing three times with PBST for 5 min each and twice with PBS for 5 min each, membranes were developed with DAB and washed in water used to stop the reaction. Alternatively, gels were stained with Coomassie Blue R250.

### 2.6. Recombinant NP Purification

HEK293T cells were seeded in sixteen 10-cm diameter dishes at 5 × 10^6^ cells per dish in 25 mL complete medium 16–18 h prior to transfection. Per plate, 105 μL puma2 MARV NP or puma2 EBOV NP plasmid Qiagen miniprep (100 ng μL^−1^) and 41 μL linear PEI (1 mg mL^−1^, pH 7.0) were combined and equilibrated for 20 min at room temperature in 2.5 mL serum-free DMEM prior to being carefully added to the medium. Cells were collected 48 h post-transfection by trypsinisation in 4 mL trypsin–EDTA solution (Sigma) with 2-plates worth of cells combined into 50-mL Falcon tubes and topped up to 50 mL with PBS. Cells were pelleted at 1000 rpm for 5 min (Beckman Allegra 6R swing out rotor) washed once with PBS and repelleted. The cells were lysed in 4 mL ice-cold hypotonic buffer consisting of 20 mM HEPES pH 7.5, 5 mM KCl, 1.5 mM MgCl_2_, 1 mM DTT, 1 tablet of cOmplete™ EDTA free protease inhibitor cocktail (Roche, Nutley, NJ, USA) per 50 mL. DNA was sheared by passing through a 30-gauge needle several times on ice. Samples were microfuged in 2-mL tubes at 6000 rpm for 10 min at 4 °C (5415D microcentrifuge, Eppendorf, Hauppauge, NY, USA) and the supernatants were transferred to fresh tubes and re-centrifuged at 13,000 rpm for 10 min. Clarified samples were pooled and concentrated in two 15-mL 100 kDa cut-off Amicon centrifugal filters at 3500 rpm (Beckman Allegra 6R, swing out rotor, room temperature) until the volume was approximately 800 µL. Samples were clarified by microcentrifugation at high speed for 5 min immediately before loading 400 µL on to CsCl gradients (40–25%, 5% steps in TNE—10 mM Tris-HCl pH 7.4, 150 mM NaCl, 1mM EDTA). Gradients were centrifuged at 25,000 rpm (Beckman SW41Ti) for 18 h at 20 °C. The NP bands were collected by side-puncture with an 18-gauge needle, the samples combined and dialyzed in 10-kDa cut-off Slide-A-Lyzer cassettes (Thermo Fisher Scientific, Waltham, MA, USA) against PBS at 4 °C. Samples were quantified by micro-BCA assay and analyzed by SDS-PAGE and silver stain. Samples were made to 15% glycerol, aliquoted and flash frozen in liquid nitrogen and stored at −80 °C.

### 2.7. nluc–NP C-Terminal Domain Fusion Protein Expression and Purification

A human codon-optimized gBlocks^®^ sequence encoding the last 90 amino acids of the MLAV NP was cloned into pENCO9 [18] via *Not*I and *Hind*III to form pENCO9 nluc–MLAV NP604 with a T7 promoter driving the nluc fusion and a His_6_ tag between the nluc and NP604 region. The plasmid was mobilized to BL21(DE3) + pRARE and grown overnight in 50 mL starter cultures of TB with 2% glucose, ampicillin at 200 µg mL^−1^ and chloramphenicol at 30 µg mL^−1^ were grown at 30 °C until saturation. Cultures were poured into 450 mL glucose free TB cultures and grown with vigorous aeration in 2500-mL Bellco baffled flasks for 3 h at 25 °C and induced for 3 h with 0.1 mM IPTG. Cultures were centrifuged and the pellets drained of excess media and stored at −80 °C until ready for bead beating (BioSpec Products). Once thawed, the pellets were resuspended in 20 mL 1× IMAC plus a complete protease inhibitor tablet (Roche, Basel, Switzerland) and added to a 15-mL chamber filled halfway with 0.1-mm glass beads. The chamber was topped off with 1× IMAC buffer to remove any air bubbles and the cell/bead mixture was blended on ice within a 4 °C fridge for a total of 12 min with 2 min on and 2 min off cooling on ice in between. Once the contents settled, the cell debris was transferred to a 50-mL conical tube and centrifuged at 3000 rpm for 15 min at 4 °C (Beckman Allegra 6R, swing out). The supernatant was decanted into a new 50-mL tube and centrifuged at 9500 rpm for 15 min at 4 °C (Sorvall RC 6+, F13 FiberLite rotor). The supernatant was filtered through a 32-mm diameter 0.8/0.2-micron filter (Pall, Port Washington, NY, USA) and applied to a 1-mL HisTrapHP column equilibrated in 1× IMAC. The protein was eluted with a 0–500-mM imidazole gradient in 1× IMAC buffer. The fractions were pooled, concentrated to 800 µL and purified with 4 runs on a Superdex Increase 10/300 GL-column in PBS. The protein was pooled, made to 15% glycerol, aliquoted for storage at −80 °C and quantified by UV adsorption and analyzed by SDS-PAGE to assess purity.

### 2.8. ELISAs

#### 2.8.1. sdAb–APEX2 Titrations

(**a**) Full-length NP: recombinant NP of either MARV or EBOV in 100 µL PBS at 1 µg mL^−1^ were used to coat duplicate wells of ELISA plates o/n at 4 °C. Plates were washed three times with PBS and each well was blocked to brimming with Bioplex buffer (Bb- 2% BSA, 0.05 % Tween-20 in PBS) for an hour. Wells were then probed with 100 µL sdAb–APEX2 fusion protein dilutions in Bb for 1 h. Then probe was removed and plates were washed by filling to brimming three times with PBST and two times with PBS. Signals were developed with 100 µL 50 mM phosphate buffer pH 7.4 containing 250 µM Amplex^®^UltraRed and 2 mM H_2_O_2_ for 20 min, followed by the addition of 25 µL Amplex Red/UltraRed stop reagent and plates were read in a model 680 ELISA plate reader (BioRad, Hercules, CA, USA). Titrations were repeated once with the final plots representing the mean of two experiments and the error bars representing +/− standard deviation (SD).

(**b**) NP-C-termini: The process was essentially the same as for NP except that the coating antigens were 1 µg mL^−1^ of recombinant nluc or nluc–NP C-terminal fusions, and signals were developed with 100 µL PBS containing 50 µM Amplex^®^UltraRed and 2 mM H_2_O_2_ for 45 min without the addition of stop solution.

#### 2.8.2. Nanoluciferase Titrations

ELISA plates were coated overnight at 4 °C with 100 µL 1 µg mL^−1^ neutravidin in PBS. Plates were washed three times with PBS and then blocked by filling to brimming with Bb for 1 h. A volume of 100 µL 100 nM anti-MARV sdAb A, B or C as BAP fusion proteins purified from pecan126 as described above was applied to duplicate wells in Bb for 1 h. Wells were washed to brimming three times with PBST and two times with PBS. Bb was added to the well to brimming for 1 h to further block the sdAb and then dilutions of nluc, nluc–MARV–NP600 or nluc–MLAV–NP604 in MPBS were added to duplicate wells for 1 h. Following washing, wells were developed with injection of coelenterazine (NanoLight™ Technology, Pinetop, AZ, USA) in lucky buffer (10 mM Tris, 1 mM EDTA, 500 mM NaCl, pH 7.4) and emissions collected using a luminometer (Turner Biosystems, Sunnyvale, CA, USA) with a 2 s integration. The experiment was repeated two more times and curves are the plots of three mean RLU of nluc–NP minus the corresponding mean of the nluc alone with error bars representing +/− SD. The EC_50_ *y* value was calculated for curves that plateaued using the equation [RLU_min_ + (RLU_max_ − RLU_min_)/2]. The corresponding *x* values were calculated using one observed point greater and one less than the *y* EC_50_ using the trend function in Excel and the three values averaged and presented +/− SD nM.

### 2.9. Cytochemical Staining of Virus Infected Cells Using sdAb–APEX2 Fusion Proteins

Live virus experiments were performed at the Texas Biomedical Research Institute BSL-4 laboratory following all federal guidelines as part of the CDC Select Agent Program and with local Biohazard and Safety Committee approval. Vero-E6 cells (ATCC) were seeded at approx. 5 × 10^4^ in 100 µL complete DMEM per well in 96-well cell culture plates to be confluent 24 h later. Wells were washed once with serum-free medium before approx. 2 × 10^4^ pfu in 100 µL virus stocks of MARV Musoke and EBOV Kikwit previously described [13,14] were added within the BSL-4 laboratory. Infection was allowed to proceed for 1 h and then an equivalent volume of DMEM plus 10% FCS was added and plates were left at 37 °C with 5% CO_2_ and humidity for 24 h. Plates were immersed in 10% buffered formalin for 18 h at 4 °C and removed from the BSL-4 laboratory for probing with dimeric sdAb–APEX2 fusions and either DAB or Amplex^®^UltraRed staining as above.

## 3. Results

### 3.1. Production and Purification of the sdAb–APEX2 Fusion Proteins

**Figure 1:**
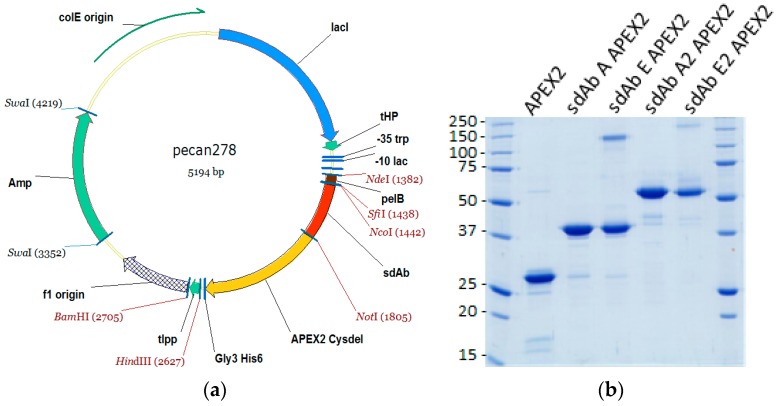
(**a**) Parental sdAb–APEX2 fusion protein expression vector pecan278 enabling switching out of protein-based affinity reagents via *Sfi*I/*Nco*I and *Not*I sites. *Amp*, ampicillin resistance gene; *lacI*, lac repressor gene; tHP, terminator; -35 trp and -10 lac, tac promoter; *pelB*, pectate lyase signal sequence; sdAb, single-domain antibody; APEX2 Cysdel, APEX2 with Cys>Ser; Gly3His6, polyhistidine tag; t*lpp*, terminator. (**b**) Coomassie Blue stained 12% SDS-PAGE gel of 5 µg of each of the unfused APEX2 and sdAb–APEX2 fusion protein preparations. Molecular weights of markers are indicated to the left in kDa.

We modified an existing periplasmic expression vector to produce unfused APEX2 and then sdAb–APEX2 fusions with a schematic of the primary construct shown in Figure 1a. A single surface-exposed thiol group encoded by cysteine 32 was switched out for hydroxyl encoded by serine to reduce the chances of inadvertent crosslinking with sdAb cysteines during folding in the oxidizing environment of the periplasm. Volumes of 500 mL cultures of the different plasmids were processed for osmotic shocking which is a simple hypertonic resuspension designed to release the periplasmic contents and affords a quick initial purification from the majority of proteins in the cytosol. Following batch IMAC purification, SEC and quantification by UV adsorption we analyzed a portion by SDS-PAGE (Figure 1b).

APEX2 and the sdAb–APEX2 fusions are well expressed and are by far the most prominent species, though they are not as clean as we are used to when expressing sdAb alone, which have generated high quality crystals after just IMAC and SEC. Importantly, the expressions only required haem supplements and were not dependent on the provision of chaperones and folding catalysts, though the optimization of these and host strains may well improve future yields and purities.

Remarkably, although the unfused sdAb E monomer is typically prone to precipitation during concentrations before SEC, we observed no such problems as an APEX2 fusion, suggesting the peroxidase has a solubilizing effect. The proteins all migrate around their expected mwt; APEX2, 29.3 kDa; sdAb A APEX2, 42.7 kDa; sdAb E APEX2, 42.1 kDa; sdAb A2 APEX2, 57.5; sdAb E2 APEX2, 56.4 kDa. Our yields from the single first expression when only collecting the peak SEC fractions are shown in Table 1 to give an idea of how many microscopy tests in chamber slides or ELISA wells can be performed. APEX2 alone yielded 6.7 mg from a 6.9 g wet weight of cells, suggesting that limits to expression level rather than purity remain in the sdAb portion of the fusion rather than APEX2.

### 3.2. sdAb–APEX2 Fusion Proteins as Immunocytochemical Staining Reagents

**Figure 2:**
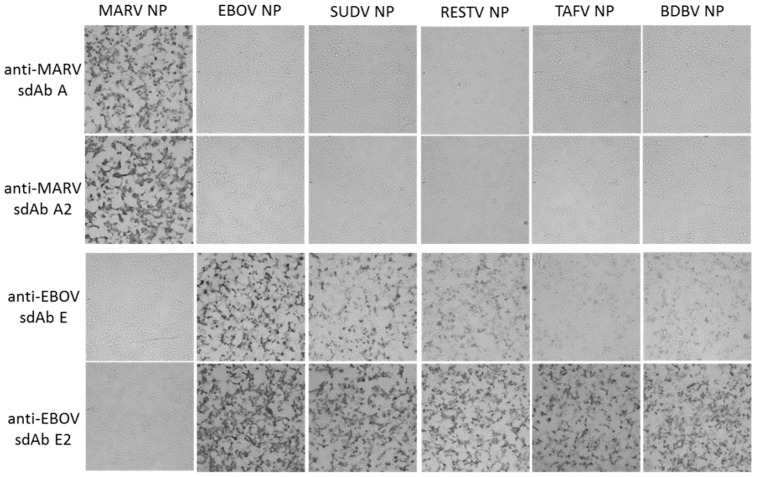
HEK293T cells were transiently transfected with various NP expression plasmids indicated at the top of the panels. At 24 h post-transfection, the cells were probed with 10 nM of the various sdAb–APEX2 fusion proteins indicated to the left of the panels, developed with 3, 3’-diaminobenzidine (DAB) for 1 min and visualized with light microscopy at 10× magnification.

**Figure 3:**
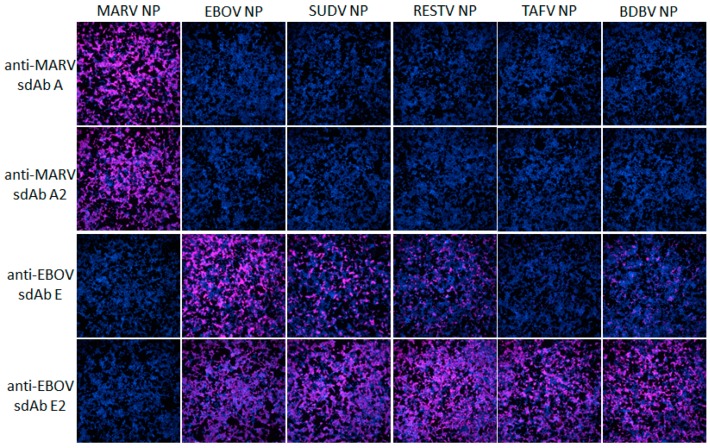
HEK293T cells were transiently transfected with various NP expression plasmids indicated at the top of the panels. At 24 h post-transfection, the cells were probed with 10 nM of the various sdAb–APEX2 fusion proteins indicated to the left of the panels, developed for 30 min with Amplex^®^Ultra Red, and visualized with fluorescence microscopy at 10× magnification.

Following the transient expression of recombinant NP genes within HEK293T cells on ibidi 8-well µ-slides, monolayers were probed with 10 nM sdAb–APEX2 fusion proteins and developed with DAB. Figure 2 shows that the anti-MARV sdAb–APEX2 fusion proteins are specific for MARV NP and not cross-reactive with any of the ebolaviral NP proteins. Conversely, the anti-EBOV sdAb–APEX2 fusions are specific for the ebolaviral NP proteins with the dimeric sdAb showing enhanced reactivity over the monomeric sdAb as we move from the cognate EBOV NP to more evolutionary/geographically distant NPs such as Sudan virus (SUDV), Reston virus (RESTV), Taï Forest virus (TAFV) and Bundibugyo virus (BDBV). We repeated the HEK293T probing but developed with Amplex^®^ UltraRed to visualize NPs using fluorescence microscopy to reveal the trend of enhanced reactivity by dimeric anti-EBOV sdAb–APEX2 over monomeric fusion is still evident (Figure 3).

### 3.3. Anti-EBOV sdAb–APEX2 Fusions as Western Blotting and ELISA Probes

**Figure 4:**
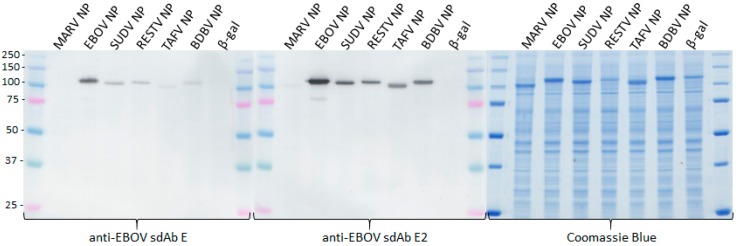
Western blotting of HEK293T cell lysates 24 h after transfecting plasmids encoding various NP genes or a control β-galactosidase gene. Blots were probed with 10 nM monomeric anti-EBOV sdAb–APEX2 or dimeric anti-EBOV sdAb–APEX2. Coomassie-stained gel of the equivalent sample loading was used for the blots. Gels were 10% SDS-PAGE and molecular weights of markers are indicated to the left in kDa.

Previously, we had assembled the anti-EBOV sdAb E as alkaline phosphatase fusion proteins and used these as probes for Western blotting of viral NP and recombinant NP lysates from both HEK293T cells and *E. coli* [14]. In these studies, using chemiluminescent substrates, NP was specifically recognized without background binding to other viral proteins or host proteins. While APEX2 does not yet appear to have a chemiluminescent substrate yet available, using DAB enables the specific recognition of NPs within blotted cell lysates (Figure 4). As with DAB-based cytochemical staining, the dimeric sdAb–APEX2 probe appears more reactive than monomeric sdAb–APEX2 probe following equivalent processing. Since the Western blot method pools multiple copies of a given protein, resolved by SDS-PAGE, in a small unit space atop an Immobilon membrane, it is ideal at capitalizing on avidity offered by dimeric probes. Though the Coomassie-stained gel shows some variation in expression levels among the recombinant NP, the dimeric sdAb–APEX2 probe again shows enhanced capacity at recognizing NPs other than that on which it was originally selected.

**Figure 5:**
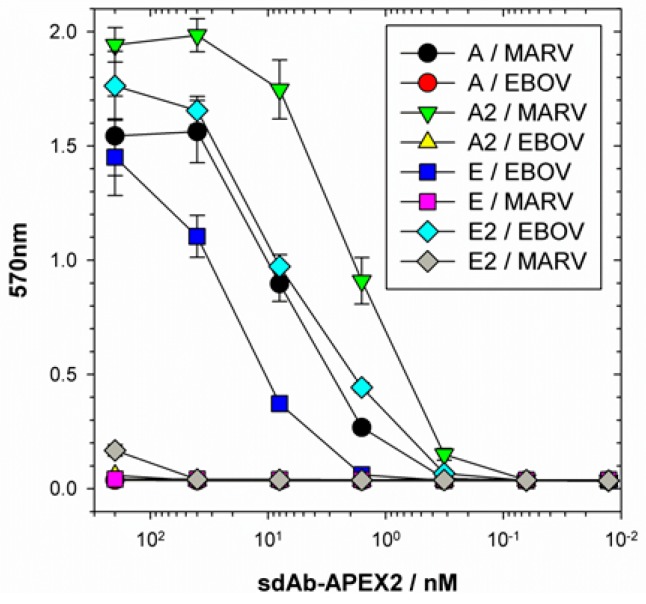
ELISA titration of sdAb–APEX2 monomers (A and E) or dimers (A2 and E2) over purified recombinant MARV or EBOV NP using Amplex^®^UltraRed substrate and colorimetric recording. Curves represent an *n* of 2 with error bars +/− SD.

In order to provide a more quantitative assessment of how much better the dimeric constructs were than the monomeric constructs, we employed APEX2’s ability to utilize Amplex^®^UltraRed ultra in a colorimetric ELISA titration of the sdAb–APEX2 fusions over a constant amount of purified recombinant NP (Figure 5).

Using one absorbance unit as our mid-point on the linear portions of the curves, it appears that the dimeric sdAb–APEX2 fusions require 4- to 5-fold less protein to generate an equivalent signal compared to the monomeric fusions. The ability to generate large amounts of the anti-EBOV sdAb E fused to a monomeric reporter has given us the ability to quantify the improvement of the dimer over the monomer since insolubility issues had previously dramatically skewed enhancement in ELISAs that just employed sdAb constructs (Figure 2e of [19]).

### 3.4. Visualization of MLAV NP by anti-MARV sdAb–APEX2 Fusions

**Figure 6:**
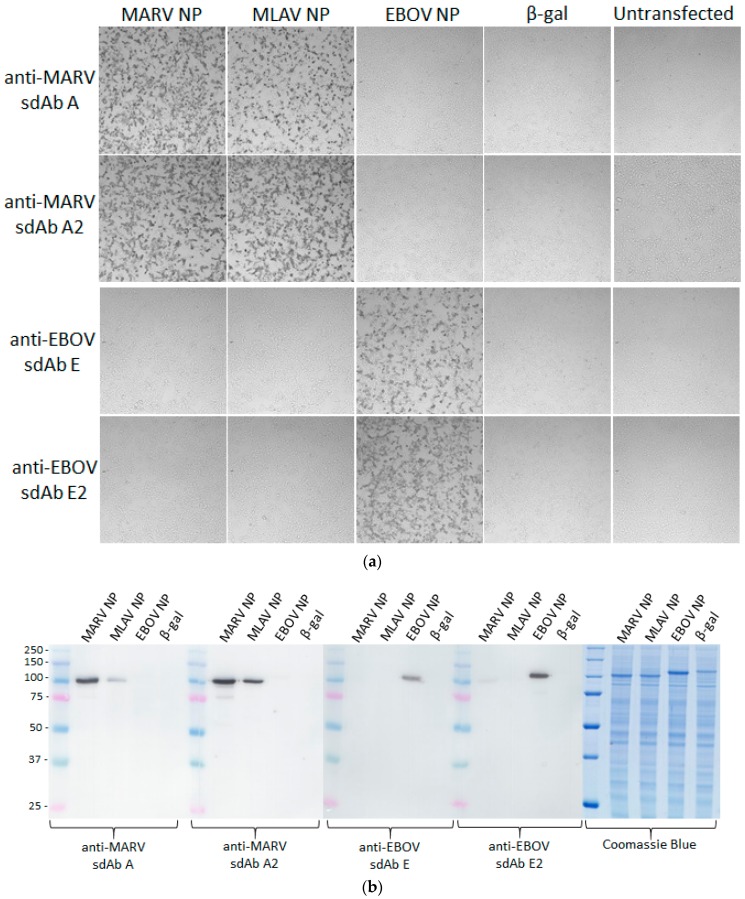

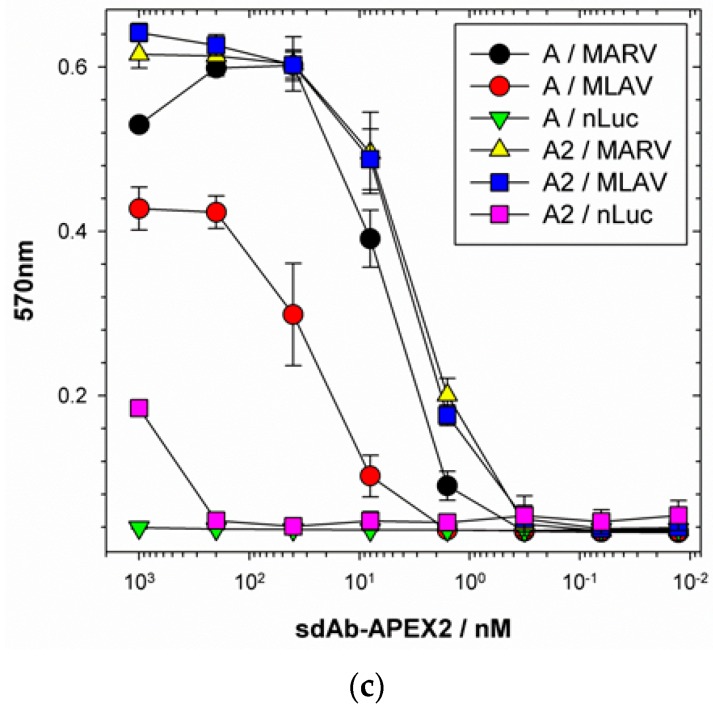
(**a**) DAB staining of HEK293T cells following the transient expression of either MARV, MLAV or EBOV NP genes followed by probing with 10 nM either monomeric (sdAb A and sdAb E) or dimeric (sdAb A2 and sdAb E2) APEX2 fusion proteins. Negative controls were β-galactosidase plasmid transfection and untransfected cells and magnification was at 10×. (**b**) DAB developed Western blots of HEK293T cell lysates after the transient expression of the MARV, MLAV, EBOV NP genes, or a control β-galactosidase gene and probing with 10 nM of either monomeric (sdAb A and sdAb E2) or dimeric (sdAb A2 and sdAb E2) APEX2 fusion proteins. Coomassie-stained 12% gel of the lysates to show equivalent loading. Molecular weights of markers are indicated to the left in kDa. (**c**) Amplex^®^UltraRed ELISA titration of monomeric versus dimeric anti-MARV sdAb–APEX2 fusions over immobilized nluc–NP C-terminal domain fusions of MARV or MLAV. Curves represent an n of 2 with error bars +/− SD.

Transient transfection of a human codon-optimized NP gene encoded by the MLAV genome was used to explore the recognition capacity of anti-MARV sdAb–APEX2 fusions in cell staining and Western blotting. Figure 6a,b show that both the monomeric and dimeric sdAb constructs can detect the MLAV NP in both assay formats with the dimeric sdAb–APEX2 probe showing enhanced recognition capacity over monomeric sdAb–APEX2. Anti-EBOV sdAb–APEX2 as a dimer but not a monomer displays very weak recognition of the MARV NP by Western blot, as also faintly seen in Figure 4, but not MLAV NP. Neither monomeric nor dimeric anti-EBOV sdAb detect MARV or MLAV NP by cell staining. Coomassie staining of the lysates reveals a little less MLAV NP is produced than MARV NP, which complicates an accurate side-by-side comparison. We therefore employed the anti-MARV sdAb–APEX2 fusions in ELISA titrations on passively immobilized purified nluc (negative control) and nluc–NP C-terminal fusions to give us a semi-quantitative idea of how much more effective the anti-MARV sdAb dimers are at binding equivalent amounts of MLAV versus MARV (Figure 6c).

Using 0.25 absorbance units as our mid-point on the linear portions of the curves indicates that the dimer is essentially showing parity between MARV and MLAV, whereas approx. 10-fold more monomer is required to generate the equivalent signal on MLAV as opposed to MARV.

### 3.5. Monovalent Anti-MARV sdAb Binding of Nanoluciferase MARV/MLAV–NP Fusions

**Figure 7:**
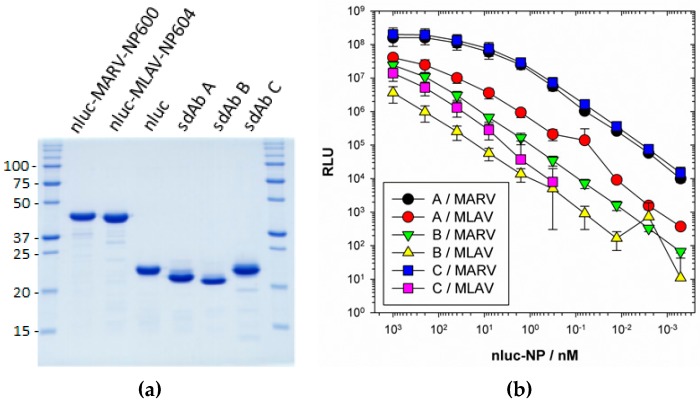
(**a**) Coomassie-stained 12% SDS-PAGE gel of 10 µg of the monovalent binding assay component proteins; sdAb, nluc and nluc–NP C-terminal domain. Molecular weights of markers are indicated to the left in kDa. (**b**) Titrations of fusions of nluc–NP C-terminal domains of either MARV or MLAV over oriented sdAb A, B or C with curves representing an n of 3 with error bars +/− SD. EC_50_ values +/− SD calculated for MARV were; sdAb A, 21.5 +/− 3.0 nM; sdAb B, 274.7 +/− 39.5 nM; sdAb C, 21.0 +/− 2.5 nM. These compare favorably with our previous determinations using this assay format; sdAb A, 24.7 +/− 6.1 nM; sdAb B, 131.6 +/− 33.1 nM; sdAb C, 32.0 +/− 1.0 nM [18].

Emboldened by our ability to detect NPs encoded by the sole member of a novel genus within the family *Filoviridae* by one of our sdAb as an APEX2 fusion, we sought more insight into the interaction of this and another two anti-MARV NP sdAb with MLAV NP. We first employed monovalent interaction assays, where monomeric nanoluciferase fusions of the NP C-terminus were titrated across monomeric oriented sdAb [18]. Nanoluciferase enables the chemiluminescent substrate coelenterazine to be employed, allowing a 9–10 log dynamic range, which is superior to what we could achieve with APEX2 and Amplex^®^UltraRed substrate. Figure 7a shows Coomassie-stained SDS preparations of the assay component proteins showing high purity. Figure 7b shows the titrations, revealing MLAV probing cannot plateau at the maximum concentration of nluc–NP tracer compared to MARV. Despite the inability to calculate EC_50_ values for MLAV, if we employ 1 × 10^6^ RLU as a linear comparator amongst the curves, we see that sdAb A and B are around 20-fold less effective on MLAV over MARV, while sdAb C exhibits around a 1000-fold decrease.

### 3.6. Rationalizing Decreases in Anti-MARV sdAb Potency for MLAV NP

**Figure 8:**
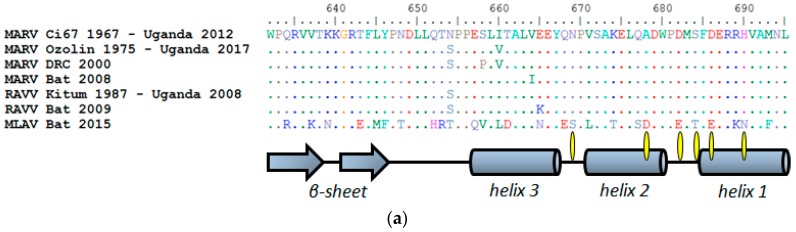

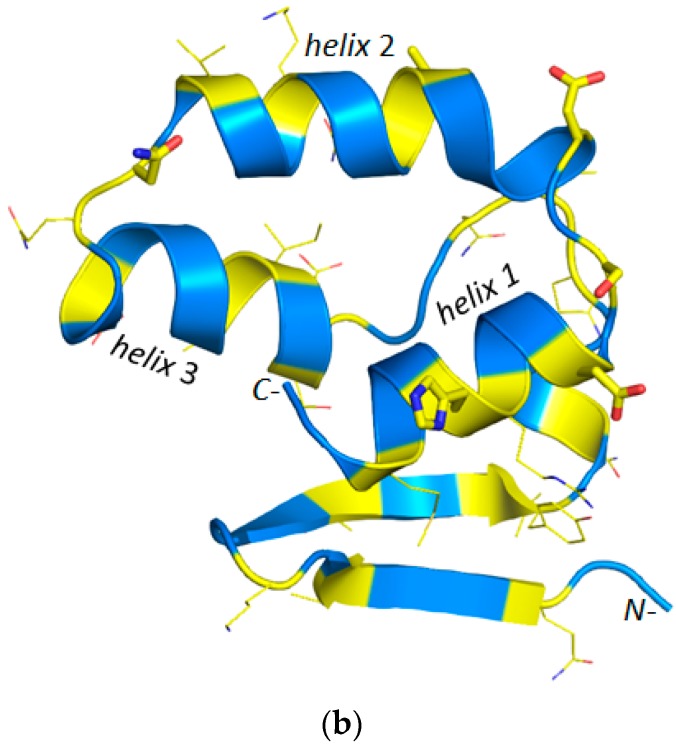
(**a**) Alignment of amino acid sequences of representative naturally occurring human or bat MARV and RAVV NP C-termini with date ranges of sampling, with the Kween MARV outbreak in 2017 [24] and MLAV [20] updating Figure 5a of [18]. Shown below are the secondary structures occurring in our Musoke crystal structure. Numbering uses the MARV/RAVV scheme since MLAV has two extra amino-acids elsewhere in NP. Mutations predicted to directly impact sdAb contacts are indicated with yellow ovals. MARV Musoke 1980, Angola 2005, and Leiden 2008 are equivalent to Ci67 in the region of NP shown (i.e., residues 632–695). (**b**) Pymol [25] rendering of a cartoon of the MARV Musoke NP C-terminal domain as bound by sdAb A (PDB ID: 6APP_B), with residues predicted to mutate between MARV and MLAV shown in yellow as lines (non-sdAb contacts) or sticks (contacts).

Examination of the primary amino-acid sequence of the MLAV NP C-terminus indicates strong potential for secondary structural conservation with MARV and RAVV (Figure 8a). Residues capable of forming three alpha-helices are at the extreme end of NPs rather than occurring as two helices distal from the C-terminal end as occurs in ebolaviruses. Indeed, phylogenetic analysis partitions the genome within a new Filovirus genus, *Dianlovirus*, sharing a more recent common ancestor with the genus *Marburgvirus* than with the genus *Ebolavirus* [20]. Based on this high sequence homology, and that two of our sdAb still retain a reasonable 1/20^th^ of their monovalent affinity for the MLAV NP C-terminus, we would expect an overall tertiary architecture very similar to our existing MARV Musoke structure. Figure 8b shows a cartoon of the Musoke structure with all of the side-chains prone to mutate between MARV and MLAV highlighted.

**Figure 9:**
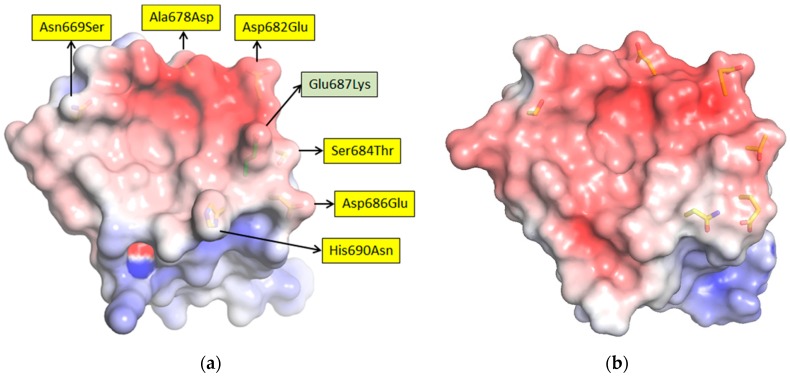
(**a**) Pymol rendering of the electrostatic surface potential of the Musoke NP C-terminal domain with scale ranging from −5 (red) to +5 (blue) K_b_T/e_c_. Side-chains of predicted sdAb contacts mutated in the MLAV NP are shown as sticks, with arrows to yellow labels indicating the changes. The arrow to green label highlights a mutation occurring in two laboratory mouse-adapted MARV Ci67 isolates. (**b**) Electrostatic surface potential of an I-TASSER generated model of MLAV NP C-terminal domain based upon a template of PDB ID 6APP_B with the sdAb contact mutations shown as sticks.

The MLAV NP C-terminus has 27 amino acid differences relative to MARV Musoke; though since the side-chains are on the outer surfaces rather than the interior, the mutations are unlikely to impact the overall packing of the helices. Therefore, the hydrophobic basin which hosts the main drivers of affinity, hydrophobic amino acids of each sdAb paratope, and the negatively charged overlook that offers salt-bridging opportunities, would likely be retained in some form. Figure 9a shows the electrostatic surface potential of the Musoke structure with sdAb contacts prone to mutate highlighted. Iterative threading assembly refinement (I-TASSER [26]) of the last 63 residues of MLAV NP yielded a model with a confidence score of +1.48 (where the range of best to worst is +2 to −5) possessing a similar overall landscape to MARV (Figure 9b). Conservation usually indicates a critical function, and that this architectural feature appears preserved from African MARV to Chinese MLAV would imply a vital role in the viral replication cycle. Human and bat MARV and RAVV isolates conserve the overlook and basin with mutations occurring off to the side of the C-terminal domain (Figure 5 of [18]), a feature MLAV also exhibits with N654T, S658V and I660L. Intriguingly, MARV Ci67 isolates adapted to immunocompetent mice (GQ433351) or *scid* mice (GQ433352), exhibit a charge reversal in the overlook (Glu687Lys) [27], though it is not definitively known whether the mutation contributes to host adaptation. Such variation, along with the MLAV mutations, indicates potential for plasticity in the make-up of the overlook not previously observed in nature (e.g., Figure 8) or noted during laboratory adaptation [28].

**Figure 10:**
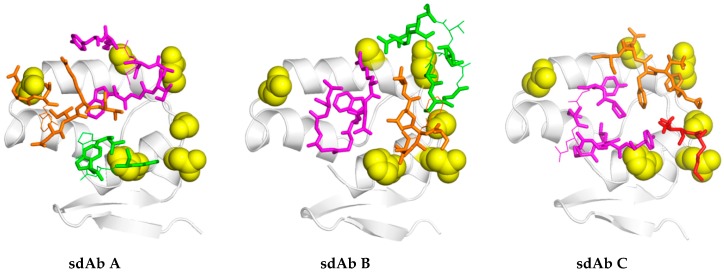
Top-down view of the epitope for each sdAb with MARV > MLAV variable residue side-chains predicted to be major contacts shown as yellow spheres. Only the CDR loops are shown as sticks (contacts) or lines (non-contacts) and colored; CDR1, orange; CDR2, green; CDR3, purple. In the sdAb C complex framework 1 (FR1) contacts are shown in red which for chains E and F involve Lys1 and Val2 though in chains A and B only involves Lys1 *via* non-bonded contacts to Glu687 and Asp686.

Six amino-acid mutations may directly impact sdAb binding; four are conservative (Asp682Glu, Ser684Thr, Asp686Glu and His690Asn) and two are not (Asn669Ser and Ala678Asp). The altered landscape of MLAV NP would likely hinder access to sdAb with footprints towards the closed end of the basin (sdAb B and C) rather the open aspect (sdAb A) as shown in (Figure 10). Though Asn669Ser is non-conservative, the mutation would result in a smaller side-chain unlikely to cause a large disruption for sdAb A. However, mutations at 682, 684 and 686, though conservative in terms of the reactive charges, are extended by a CH_2_ group which may reshape the overlook and compromise the ability of sdAb B and C to simultaneously engage both the basin and overlook. Perhaps sdAb B is less impacted than sdAb C owing to the high degree of flexibility in the triple Gly CDR3 loop which may enable an altered approach to binding the basin, and/or that the dramatic restructuring of sdAb C CDR1 loop for binding [18] is thwarted by the MLAV mutations.

**Figure 11:**
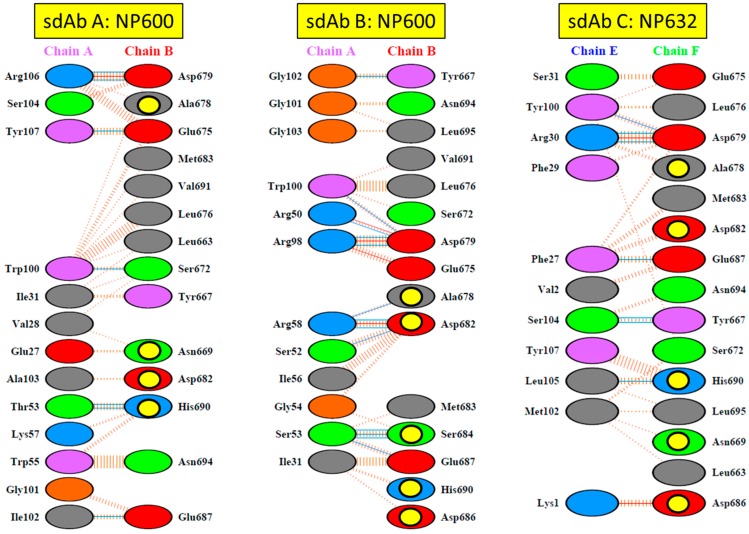
Schematic interaction maps of the three anti-MARV sdAb with the MARV Musoke NP C-terminus based on analysis of sdAb A/NP600 (PDB ID: 6APP), sdAb B/NP600 (PDB ID: 4W2O), sdAb C/NP632 (PDB ID: 4W2Q) using PDBSum. Residues are labelled as follows; Positive = His, Lys, Arg; negative = Asp, Glu; neutral = Ser, Thr, Asn, Gln; aliphatic = Ala, Val, Leu, Ile, Met; aromatic = Phe, Tyr, Trp; Pro, Gly. Interactions are labelled as follows; salt bridges, red lines; hydrogen bonds, blue lines; non-bonded contacts, striped brown lines with the width of the lines proportional to the number of atomic contacts. The yellow circles highlight those residues that are mutated in the MLAV NP.

Reference to interaction maps generated through PDBSum [29] also reveals details of the nature of direct contacts between sdAb and NP residues that may be impacted in transitioning from MARV to MLAV (Figure 11). SdAb A appears to interact with only 4 of the 6 variable amino-acids, while sdAb B and C utilize 5. For sdAb A, only 1 of the 4 residues appears a major contact; His690Asn may impact the non-bonded contacts and two hydrogen bonds occurring with Thr53 of CDR1. For sdAb B, the double salt-bridging and hydrogen bonding between Arg58 of CDR2 on Asp682 may still occur on the bulkier extension to Glu, and Ser684Thr hydrogen bonding may likewise proceed. For sdAb C salt-bridging disruption of Lys1 with Asp686Glu might occur, though this varies between the chains analyzed in the lattice, suggesting some leeway here. Though the other interactions are predicted to be fairly minor for sdAb C, we know that this is the worst performer on Western blotting (A > B > C from Figure 1e of [18]) and may well be more conformationally sensitive to minor changes in antigen structure, also contributing to the dramatic loss in binding to MLAV.

### 3.7. Employing sdAb–APEX2 Fusions as Microscopy Probes for Virus Infections

**Figure 12:**
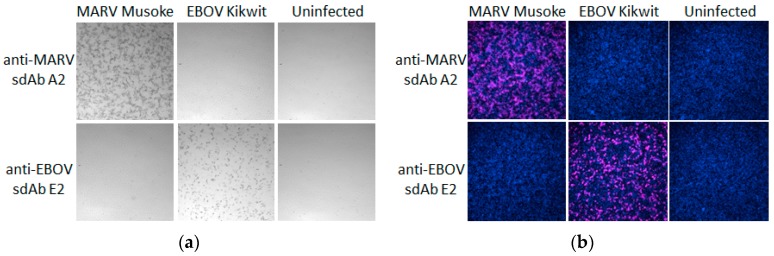
Dimeric sdAb–APEX2 fusions at 10 nM were used to probe Vero cells 24 h post-infection with either MARV Musoke or EBOV Kikwit for development using (**a**) DAB or (**b**) Amplex^®^UltraRed. Magnification was at 10×.

As proof of principle to demonstrate the APEX2 fusions were capable of detecting NPs during virus infection, we probed Vero-E6 cells in 96-well plates that had been infected with either MARV or EBOV. We employed the dimeric rather than the monomeric fusions in this assay to reveal DAB staining (Figure 12a) and Amplex^®^UltraRed staining (Figure 12b) corresponding to the specificity of the sdAb dimer employed. The assay was performed in standard 96-well culture plates that were not specifically designed for microscopy and the imaging is suboptimal for focusing well.

## 4. Discussion

APEX2 appears ideally suited as a reporter that is compatible with fusion to monomeric and dimeric sdAb targeted to the periplasm. While the cytosolic expression of APEX2 fused to the antibody binding domain of protein A was successful [30], being able to express high levels of a peroxidase in the *E. coli* periplasm immediately facilitates genetic fusion to any recombinant antibody of choice, not only specific for other filoviral proteins but any antigen of interest. In contrast, the cytosolic expression of recombinant antibody fragments, even in hosts engineered to have oxidizing cytosols to enable disulfide bond formation, can be fickle with success very much on a case-by-case basis even within the highly soluble single-domain family of antibodies [31]. Growth of *E. coli* and harvesting recombinant proteins is still probably the most straightforward and inexpensive route to generate lab-scale reagents. Immortalization of the constructs in silico ensures these are available indefinitely and are not limited by issues such as hybridoma instability and costly scale-up of tissue culture production. Often times, we note that commercially available monoclonal antibodies are incredibly costly for limited amounts of material, and there is no guarantee that a particular reagent will be available indefinitely. For example, the widely used anti-M13-HRP conjugate from GE was discontinued without reason, leaving phage display labs around the world scrambling to find alternatives.

The capacity of APEX2 to utilize DAB provides a simple route to detect antigens on blots and in cells and the fusions should be compatible as one-step probes for immunohistochemistry of tissues from infected animals where fairly complex sandwiching of primary antibodies with secondary conjugated antibodies and can often be required for demanding applications [32]. APEX2 catalysis of Amplex^®^UltraRed substrate also offers a highly sensitive fluorescence detection option without changing the actual probe and this is most evident in the virus infected cell probing (Figure 11) where equivalent concentrations of probe struggled to yield very strong cell staining by DAB. The Amplex^®^UltraRed signals are directly proportional to the amount of substrate being used (our NP ELISA utilized five-fold more substrate than our NP C-terminal domain ELISA for example, cf. Figure 5 and Figure 6c). Though one might envisage diagnostic ELISA development of the Amplex^®^UltraRed approach, the substrate cost and freezer storage requirements would currently limit studies to furnished laboratory settings. However, initial development [33] and recent evolutionary optimization [34] of a split-APEX2 format, where catalytic activity is only reconstituted when the two portions of the enzyme come close enough together may have unique diagnostic advantages. These studies expressed split-APEX2 fusions to proteins of interest intracellularly to evaluate conditions that enhance proximity in vivo. Our ability to overproduce large amounts of APEX2 bodes well for generating split-APEX2 fusions to sdAb and evaluating the ability of NP polymers to reconstitute catalytic activity in vitro. If successful, such a solution phase assay would simply require the addition of a patient sample to a pre-existing mix of sdAb–split-APEX2 fusion proteins and substrate to elicit a color change.

Our initial use of recombinant NP expression in cells and from lysates for pathfinding, revealed a striking ability of the anti-EBOV dimer to compensate for weaknesses of the monomer in binding NPs that were distant from the original virus used for selection. Originally the sdAb E monomer was known to bind the different viruses in the order ZEBOV, SUDV, RESTV, TAFV with TAFV = BDGV on Western blots [14]. Here, the dimers’ 5-fold enhancement over the monomers shown in Figure 5 has increased the cross-reactivity among the various strains in both cells and on blots. The tendency of NP to form long helices of several hundred monomers makes it an ideal antigen to capitalize on avidity of the dimeric sdAb–APEX2 probes. Avidity can be thought of as an apparent increase in affinity resulting from the decreased frequency that both paratopes on dimeric sdAb–APEX2 will simultaneously dissociate in a given time-frame compared with the single paratope of monomeric sdAb–APEX2. NP polymers are known to be expressed in puncta, akin to virus factories, which will further serve to localize sdAb epitopes in cytochemical assays. Since the NP C-terminal epitopes of our sdAb are expected to be repetitively displayed along the length of the ribonucleoprotein [35,36], each of the sdAb–APEX2 dimers may well be capable of interacting with epitopes within the same RNP or even different RNPs. Thus, affinity decreases for monomeric sdAb binding distantly related NP with less shape and charge complementarity, can be negated to a large extent through a simple dimerization step owing to the very high antigen density in this type of assay.

We extended the avidity advantage to the recognition of MLAV by our anti-MARV sdAb. Though monomeric analysis showed an approximate 20-fold decrease in binding MLAV (Figure 6c and Figure 7b), the dimer exhibited negligible difference in binding a high-density coat of the C-termini (Figure 6c). That our sdAb still recognize MLAV suggests the NP C-terminus shares an overall architecture that is similar to MARV, as also indicated by molecular modelling, and it will be interesting to determine a crystal structure to confirm this. Despite our best efforts, we have been unable to crystallize the MARV NP C-terminus without the assistance of sdAb chaperones and are curious to see what the unoccupied basin actually looks like. The propensity to form high quality crystals is empirical at best, and it may well be that the MLAV structure is sufficiently different to solve this conundrum. Though MLAV is not known to be a human pathogen, and the virus has yet to be isolated, the presence of genome in Chinese *Rousettus* bats [20] very similar to African *Rousettus*, the natural reservoir host of MARV [37] may well be a siren call for spillover. It would be tempting to speculate that the mutations between MARV and MLAV, in a region exhibiting plasticity during adaptation of MARV to mouse, are an indication of different host/environmental selective pressures on MLAV. Discovery of strong anti-ZEBOV serum responses and genomic signatures in a previous bat survey in China [38] may also point to more ebolaviruses circulating than originally thought. It may well be that our dimeric anti-EBOV sdAb–APEX2 fusions may be capable of recognizing these variants if our findings with MARV and MLAV can be extrapolated between genera.

Our proof-of-principle use of sdAb–APEX2 fusions to detect MARV and EBOV infected cells indicates the possibility to develop a multi-day focus forming assay under semi-solid media [39]. The focus would likely provide more antigen for the DAB staining approach to improve in intensity, and also enhance the ability to titrate replication competent virus as has been done using conventional antibody HRP staining regimes in 4 days for RESTV [40] and in 7 days for several other BSL-4 agents [41]. The option of using the highly sensitive fluorescent readout conforms with long established practice of immunofluorescence focus assays in 48 h for EBOV under an overlay [42].

Antiviral sdAb have often been shown to be greatly improved in neutralization potency and breadth of reactivity when multimerized (for review see [43,44]) and some can even be protective in animal models. Multi-Fab have also been shown to have elevated anti-HIV potency over monomer and to be less susceptible to virus escape [45]. The sdAb is an artificial molecule that does not exist in nature and is derived from heavy chain-only antibodies (HCAb) of camelids [46] or cartilaginous fish [47]. HCAb are themselves dimeric, pointing towards nature’s innate leverage of avidity in targeting macromolecular repeat structures of pathogens that often vary their antigenic landscapes. In hindsight, we wonder if reverting to a parental surface of soybean ascorbate peroxidase to recapitulate the natural tendency to form non-covalent dimers [10] would offer an even simpler route to forming a dimeric sdAb construct with two enzymes per molecule. Such a route may also enable the formation of a dimer of dimeric sdAb–APEX fusions, in essence a highly avid tetrameric sdAb, which can be challenging to make in very high yields in our hands using the tandem linking of sdAb in series approach.

## Figures and Tables

**Table 1:** Unoptimized first pass yields of the sdAb–APEX2 fusions following periplasmic expression and purification from a handful of grams of wet weight *E. coli* reveal the potential for tens of thousands of small volume staining tests based on 200 µL volumes of 10 nM.

sdAb	wet wt, g	mg mL^−1^	mL	mg	µM	µL Test^−1^	Tests
**A**	5.9	2.32	2.4	5.6	54.4	0.04	65,280
**A2**	5.5	0.81	2.4	1.9	14.0	0.14	16,800
**E**	4.9	1.91	1.8	3.4	45.3	0.04	40,770
**E2**	5.2	1.05	1.8	1.9	18.6	0.11	16,740

## References

[1] Van Lith S.A., van Duijnhoven S.M., Navis A.C., Leenders W.P., Dolk E., Wennink J.W., van Nostrum C.F., van Hest J.C. (2017). Legomedicine-a versatile chemo-enzymatic approach for the preparation of targeted dual-labeled llama antibody-nanoparticle conjugates. Bioconjug. Chem..

[2] Gajhede M., Schuller D.J., Henriksen A., Smith A.T., Poulos T.L. (1997). Crystal structure of horseradish peroxidase c at 2.15 A resolution. Nat. Struct. Biol..

[3] Smith A.T., Santama N., Dacey S., Edwards M., Bray R.C., Thorneley R.N., Burke J.F. (1990). Expression of a synthetic gene for horseradish peroxidase C in *Escherichia coli* and folding and activation of the recombinant enzyme with Ca^2+^ and heme. J. Biol. Chem..

[4] Kondo A., Kohda J., Endo Y., Shiromizu T., Kurokawa Y., Nishihara K., Yanagi H., Yura T., Fukuda H. (2000). Improvement of productivity of active horseradish peroxidase in escherichia coli by coexpression of DSB proteins. J. Biosci. Bioeng..

[5] Gundinger T., Spadiut O. (2017). A comparative approach to recombinantly produce the plant enzyme horseradish peroxidase in *Escherichia coli*. J. Biotechnol..

[6] Krainer F.W., Capone S., Jager M., Vogl T., Gerstmann M., Glieder A., Herwig C., Spadiut O. (2015). Optimizing cofactor availability for the production of recombinant heme peroxidase in *Pichia pastoris*. Microb. Cell. Fact..

[7] Yamagata M., Sanes J.R. (2018). Reporter-nanobody fusions (ranbodies) as versatile, small, sensitive immunohistochemical reagents. Proc. Natl. Acad. Sci. USA.

[8] Decad G.M., Nikaido H. (1976). Outer membrane of gram-negative bacteria. Xii. Molecular-sieving function of cell wall. J. Bacteriol..

[9] Lam S.S., Martell J.D., Kamer K.J., Deerinck T.J., Ellisman M.H., Mootha V.K., Ting A.Y. (2015). Directed evolution of apex2 for electron microscopy and proximity labeling. Nat. Methods.

[10] Patterson W.R., Poulos T.L. (1995). Crystal structure of recombinant pea cytosolic ascorbate peroxidase. Biochemistry.

[11] Martell J.D., Deerinck T.J., Sancak Y., Poulos T.L., Mootha V.K., Sosinsky G.E., Ellisman M.H., Ting A.Y. (2012). Engineered ascorbate peroxidase as a genetically encoded reporter for electron microscopy. Nat. Biotechnol..

[12] Hung V., Udeshi N.D., Lam S.S., Loh K.H., Cox K.J., Pedram K., Carr S.A., Ting A.Y. (2016). Spatially resolved proteomic mapping in living cells with the engineered peroxidase apex2. Nat. Protoc..

[13] Sherwood L.J., Osborn L.E., Carrion R., Patterson J.L., Hayhurst A. (2007). Rapid assembly of sensitive antigen-capture assays for marburg virus, using in vitro selection of llama single-domain antibodies, at biosafety level 4. J. Infect. Dis..

[14] Sherwood L.J., Hayhurst A. (2013). Ebolavirus nucleoprotein c-termini potently attract single domain antibodies enabling monoclonal affinity reagent sandwich assay (MARSA) formulation. PLoS ONE.

[15] De Genst E., Silence K., Decanniere K., Conrath K., Loris R., Kinne J., Muyldermans S., Wyns L. (2006). Molecular basis for the preferential cleft recognition by dromedary heavy-chain antibodies. Proc. Natl. Acad. Sci. USA.

[16] Stijlemans B., Conrath K., Cortez-Retamozo V., Van Xong H., Wyns L., Senter P., Revets H., de Baetselier P., Muyldermans S., Magez S. (2004). Efficient targeting of conserved cryptic epitopes of infectious agents by single domain antibodies. African trypanosomes as paradigm. J. Biol. Chem..

[17] Strauss M., Schotte L., Thys B., Filman D.J., Hogle J.M. (2016). Five of five vhhs neutralizing poliovirus bind the receptor-binding site. J. Virol..

[18] Garza J.A., Taylor A.B., Sherwood L.J., Hart P.J., Hayhurst A. (2017). Unveiling a drift resistant cryptotope within marburgvirus nucleoprotein recognized by llama single-domain antibodies. Front. Immunol..

[19] Darling T.L., Sherwood L.J., Hayhurst A. (2017). Intracellular crosslinking of filoviral nucleoproteins with xintrabodies restricts viral packaging. Front. Immunol..

[20] Yang X.L., Tan C.W., Anderson D.E., Jiang R.D., Li B., Zhang W., Zhu Y., Lim X.F., Zhou P., Liu X.L. (2019). Characterization of a filovirus (mengla virus) from rousettus bats in china. Nat. Microbiol..

[21] Sherwood L.J., Hayhurst A. (2012). Hapten mediated display and pairing of recombinant antibodies accelerates assay assembly for biothreat countermeasures. Sci. Rep..

[22] Neu H.C., Heppel L.A. (1965). The release of enzymes from *Escherichia coli* by osmotic shock and during the formation of spheroplasts. J. Biol. Chem..

[23] Zanta-Boussif M.A., Charrier S., Brice-Ouzet A., Martin S., Opolon P., Thrasher A.J., Hope T.J., Galy A. (2009). Validation of a mutated pre sequence allowing high and sustained transgene expression while abrogating WHV-X protein synthesis: Application to the gene therapy of was. Gene. Ther..

[24] Nyakarahuka L., Shoemaker T.R., Balinandi S., Chemos G., Kwesiga B., Mulei S., Kyondo J., Tumusiime A., Kofman A., Masiira B. (2019). Marburg virus disease outbreak in kween district Uganda, 2017: Epidemiological and laboratory findings. PLoS Negl. Trop. Dis..

[25] DeLano W.L. (2010). The Pymol Molecular Graphics System.

[26] Yang J., Zhang Y. (2015). I-tasser server: New development for protein structure and function predictions. Nucleic Acids Res..

[27] Lofts L.L., Ibrahim M.S., Negley D.L., Hevey M.C., Schmaljohn A.L. (2007). Genomic differences between guinea pig lethal and nonlethal marburg virus variants. J. Infect. Dis..

[28] Banadyga L., Dolan M.A., Ebihara H. (2016). Rodent-adapted filoviruses and the molecular basis of pathogenesis. J. Mol. Biol..

[29] Laskowski R.A., Jablonska J., Pravda L., Varekova R.S., Thornton J.M. (2018). Pdbsum: Structural summaries of pdb entries. Protein Sci..

[30] Lee J., Song E.K., Bae Y., Min J., Rhee H.W., Park T.J., Kim M., Kang S. (2015). An enhanced ascorbate peroxidase 2/antibody-binding domain fusion protein (apex2-abd) as a recombinant target-specific signal amplifier. Chem. Commun. (Camb.).

[31] Olichon A., Surrey T. (2007). Selection of genetically encoded fluorescent single domain antibodies engineered for efficient expression in escherichia coli. J. Biol. Chem..

[32] Perry D.L., Huzella L.M., Bernbaum J.G., Holbrook M.R., Jahrling P.B., Hagen K.R., Schnell M.J., Johnson R.F. (2018). Ebola virus localization in the macaque reproductive tract during acute ebola virus disease. Am. J. Pathol..

[33] Xue M., Hou J., Wang L., Cheng D., Lu J., Zheng L., Xu T. (2017). Optimizing the fragment complementation of APEX2 for detection of specific protein-protein interactions in live cells. Sci. Rep..

[34] Han Y., Branon T.C., Martell J.D., Boassa D., Shechner D., Ellisman M.H., Ting A. (2019). Directed evolution of split apex2 peroxidase. ACS Chem Biol.

[35] Bharat T.A., Noda T., Riches J.D., Kraehling V., Kolesnikova L., Becker S., Kawaoka Y., Briggs J.A. (2012). Structural dissection of ebola virus and its assembly determinants using cryo-electron tomography. Proc. Natl. Acad. Sci. USA.

[36] Bharat T.A., Riches J.D., Kolesnikova L., Welsch S., Krahling V., Davey N., Parsy M.L., Becker S., Briggs J.A. (2011). Cryo-electron tomography of marburg virus particles and their morphogenesis within infected cells. PLoS Biol..

[37] Towner J.S., Amman B.R., Sealy T.K., Carroll S.A., Comer J.A., Kemp A., Swanepoel R., Paddock C.D., Balinandi S., Khristova M.L. (2009). Isolation of genetically diverse marburg viruses from egyptian fruit bats. PLoS Pathog..

[38] Yang X.L., Zhang Y.Z., Jiang R.D., Guo H., Zhang W., Li B., Wang N., Wang L., Waruhiu C., Zhou J.H. (2017). Genetically diverse filoviruses in rousettus and eonycteris spp. Bats, China, 2009 and 2015. Emerg. Infect. Dis..

[39] Matrosovich M., Matrosovich T., Garten W., Klenk H.D. (2006). New low-viscosity overlay medium for viral plaque assays. Virol. J..

[40] Pickering B.S., Collignon B., Smith G., Marszal P., Kobinger G., Weingartl H.M. (2018). Detection of zaire ebolavirus in swine: Assay development and optimization. Transbound. Emerg. Dis..

[41] Ackermann-Gaumann R., Siegrist D., Zust R., Signer J., Lenz N., Engler O. (2019). Standardized focus assay protocol for biosafety level four viruses. J. Virol. Methods.

[42] Truant A.L., Regnery R.L., Kiley M.P. (1983). Development of an immunofluorescence focus assay for ebola virus. J. Clin. Microbiol..

[43] Vanlandschoot P., Stortelers C., Beirnaert E., Ibanez L.I., Schepens B., Depla E., Saelens X. (2011). Nanobodies^®^: New ammunition to battle viruses. Antiviral Res..

[44] Wu Y., Jiang S., Ying T. (2017). Single-domain antibodies as therapeutics against human viral diseases. Front. Immunol..

[45] Galimidi R.P., Klein J.S., Politzer M.S., Bai S., Seaman M.S., Nussenzweig M.C., West A.P., Bjorkman P.J. (2015). Intra-spike crosslinking overcomes antibody evasion by HIV-1. Cell.

[46] Hamers-Casterman C., Atarhouch T., Muyldermans S., Robinson G., Hamers C., Songa E.B., Bendahman N., Hamers R. (1993). Naturally occurring antibodies devoid of light chains. Nature.

[47] Greenberg A.S., Avila D., Hughes M., Hughes A., McKinney E.C., Flajnik M.F. (1995). A new antigen receptor gene family that undergoes rearrangement and extensive somatic diversification in sharks. Nature.

